# Low Density Solvent-Based Dispersive Liquid-Liquid Microextraction for the Determination of Synthetic Antioxidants in Beverages by High-Performance Liquid Chromatography

**DOI:** 10.1155/2013/414398

**Published:** 2013-06-17

**Authors:** Hasan Çabuk, Mustafa Köktürk

**Affiliations:** Department of Chemistry, Faculty of Arts and Sciences, Bülent Ecevit University, 67100 Zonguldak, Turkey

## Abstract

A simple and efficient method was established for the determination of synthetic antioxidants in beverages by using dispersive liquid-liquid microextraction combined with high-performance liquid chromatography with ultraviolet detection. Butylated hydroxy toluene, butylated hydroxy anisole, and tert-butylhydroquinone were the antioxidants evaluated. Experimental parameters including extraction solvent, dispersive solvent, pH of sample solution, salt concentration, and extraction time were optimized. Under optimal conditions, the extraction recoveries ranged from 53 to 96%. Good linearity was observed by the square of correlation coefficients ranging from 0.9975 to 0.9997. The relative standard deviations ranged from 1.0 to 5.2% for all of the analytes. Limits of detection ranged from 0.85 to 2.73 ng mL^−1^. The method was successfully applied for determination of synthetic antioxidants in undiluted beverage samples with satisfactory recoveries.

## 1. Introduction

Antioxidants are widely used as preservatives in food and cosmetics to prolong the shelf life by protecting them against deterioration caused by oxidation [1]. Most of the commonly used antioxidants are synthetic compounds such as butylated hydroxy toluene (BHT), butylated hydroxy anisole (BHA), tert-butylhydroquinone (TBHQ), and propyl gallate (PG) because of their chemical stability, low cost, and availability [2]. In several countries, the use of these antioxidants is regulated by various legislating authorities such as European Union Directives and Regulations, the FDA in the United States, Food Standards Australia New Zealand for Australia and New Zealand, and Joint FAO/WHO Expert Committee on Food Additives [3]. According to Turkish Food Codex, in compliance with European Union Directives, the antioxidants mentioned above are permitted for use, individually or in combination, in oils, fats, and lipid containing foods usually at concentrations up to 100–200 *μ*g g^−1^, while their usage in beverages has been banned [4]. Although they ultimately play an important role in protecting product quality and safety, excess antioxidants added to food might cause a loss of nutrients and even produce toxic effects [5, 6]. Consequently, the analytical monitoring of these compounds in foods is of considerable importance. 

A variety of analytical methods for determining synthetic antioxidants in food, drugs, and cosmetics have been reported to date. The methods include high-performance liquid chromatography (HPLC) [6–10], gas chromatography [1, 2, 11], and micellar electrokinetic chromatography [5, 12]. HPLC with UV detection was the most common determination technique, following an adequate sample preparation step [3]. In general, extraction techniques such as extraction with solvents [7, 13, 14] and solid phase extraction (SPE) [1, 15] are used to clean up and preconcentrate the synthetic antioxidants. However, the methods previously reported usually involve large quantities of organic solvents in liquid solvent extraction and in the elution process of SPE. Recently, dispersive liquid-liquid microextraction (DLLME) has been introduced as a novel sample preparation technique, which provides high enrichment factors together with a significant reduction of organic solvent consumption as well as extraction time [16]. The basic principle of this method is the dispersion of extraction solvent assisted with a disperser solvent within an aqueous solution that generates a very high contact area between the aqueous phase and the extraction solvent [17]. Since its introduction, it has fast become one of the most popular analytical sample preparation techniques because of its advantages such as simplicity, rapidity, and low consumption of solvents and reagents. To date, DLLME has been applied for the analysis of various organic and inorganic pollutants in aqueous samples [18–22], and even in solid samples after an adequate pretreatment [23–25]. 

The aim of the present study is to assess the suitability of DLLME technique combined with HPLC-UV for the determination of three synthetic antioxidants (BHA, BHT, and TBHQ). The factors affecting the microextraction efficiency were studied in details and the optimal conditions were established. The analytical performance and possible application of the method in beverage samples were investigated. Although DLLME has been applied for the determination of BHA and BHT [9], to the best of our knowledge, this is the first reported application of DLLME for the extraction and preconcentration of TBHQ from an aqueous sample matrix.

## 2. Experimental 

### 2.1. Reagents and Solutions

All of the reagents used in the experiments were of analytical grade. Butylated hydroxy toluene (BHT) was purchased from Supelco (Bellefonte, PA, USA). Butylated hydroxy anisole (BHA) and tert-butylhydroquinone (TBHQ) were obtained from Sigma-Aldrich (Steinheim, Germany). Anhydrous Na_2_SO_4_ and HPLC grade acetonitrile were also obtained from Sigma-Aldrich (Steinheim, Germany). Acetone, methanol, 1-octanol, and 1-decanol were purchased from Merck (Darmstadt, Germany). Water was purified with a Direct-Q3 water purification system (Millipore, Bedford, MA, USA).

The stock solutions of the antioxidants (1000 *μ*g mL^−1^) were prepared by dissolving each individual standard of antioxidants in acetonitrile and stored at 4°C. Working solutions were obtained by appropriate dilution of the stock standard solutions.

Tap water samples were collected from our laboratory and analyzed without any previous treatment or filtration. Several types of marketed beverage samples including mineral water, cherry juice, apple juice, and mixed fruit juice were purchased from local supermarkets. The beverage samples were filtered through a 0.45 *μ*m filter and 5 mL portions were subjected to DLLME method without dilution before and after spiking with antioxidants at different concentrations. 

### 2.2. Instrumentation and Chromatographic Conditions

The chromatographic analysis was performed by Thermo Finnigan HPLC system (San Jose, CA, USA) consisting of a P1000 pump, a AS3000 automatic injector system, a SCM 1000 degasser, and a UV1000 UV detector. The system was controlled by a Spectra System Controller SN 4000 and a software package ChromQuest 4.0. Separation was performed by means of a Phenomenex Max-RP column (250 × 4.6 mm i.d., 4.0 *μ*m) protected by a C18 guard column (4 × 3 mm i.d., Phenomenex). A gradient elution program was optimized by using the mobile phases of acetonitrile and distilled deionized water (0.1% trifluoroacetic acid). The separation was performed at room temperature with a constant flow rate of 1.3 mL min^−1^ by employing the elution program as follows: 0–5 min acetonitrile water 75 : 25 (v/v) and then a linear gradient elution from 75% acetonitrile at 5 min to 100% acetonitrile at 20 min, followed by isocratic elution with acetonitrile for 5 min. Finally, 10 min was necessary in reestablishing the initial conditions. To obtain better sensitivity, detection wavelength was checked experimentally with a series of injections of standard solution at 270, 280, and 290 nm wavelengths. The detector response for the studied compounds was the highest at 280 nm. Therefore, 280 nm wavelength was selected for further analysis. 

### 2.3. Dispersive Liquid-Liquid Microextraction Procedure

5.0 mL of standard solution (containing 500 ng mL^−1^ of each antioxidant) or real beverage sample, previously adjusted to pH 6, was transferred into a 10 mL glass test tube. Subsequently, 0.3 g NaCl was added and the tube was shaken to dissolve NaCl. 1.0 mL methanol (as disperser) containing 90 *μ*L 1-octanol (as extraction solvent) was rapidly injected into the solution using a 1 mL syringe (Hamilton, Bonaduz, Switzerland). In this step, the extraction solvent was dispersed into the aqueous sample as very fine droplets and a cloudy solution was formed in the glass test tube. The mixture was shaken gently for a few seconds and then centrifuged for 5 min at 4000 rpm (Nuve NF 615, Ankara, Turkey). Organic solvent (1-octanol) was accumulated on the surface of aqueous phase as a small drop. After this process, a technique developed in our previous study was used for the simple and easy separation of a low density organic solvent [26]. Briefly, the organic solvent together with some little aqueous phase was pipetted by using a disposable glass Pasteur pipette. Next, the flow of the aqueous phase was stopped by successively dipping the capillary tip of the pipette into anhydrous Na_2_SO_4_. The upper organic layer was then removed with a 100 *μ*L microsyringe and 20 *μ*L of this solution was injected into the HPLC by using an automatic injector. 

### 2.4. Calculation of Enrichment Factor and Extraction Recovery

Equations (1) and (2) were applied for the calculation of enrichment factor (EF) and extraction recovery (ER), respectively. Consider the following:

(1) $$\begin{array}{c} \text{EF}=\frac{C_{\text{col}}}{C_{o}}, \end{array}$$

where *C*_col_ and *C*_*o*_ were the concentration of analyte in the collected phase and the initial concentration of analyte in the sample solution, respectively. *C*_col_ was calculated from the calibration graphs of antioxidant standard solutions in the concentration range of 0.1–200 *μ*g mL^−1^. Consider the following:

(2) $$\begin{array}{c} \text{ER}=\frac{\left(C_{\text{col}}\cdot V_{\text{col}}\right)}{\left(C_{o}\cdot V_{\text{aq}}\right)}\times 100\;=\text{EF}\times \left(\frac{V_{\text{col}}}{V_{\text{aq}}}\right)\times 100, \end{array}$$

where *V*_col_ and *V*_aq_ were the volume of the collected phase and the volume of the sample solution, respectively.

## 3. Results and Discussion

In order to obtain the maximal extraction efficiency, various parameters that affect the DLLME performance and efficiency, such as the kind and the volume of the extraction and the disperser solvents, the ionic strength, pH of the aqueous samples, and the extraction time were investigated in detail. These parameters were investigated and, then, the optimal conditions were selected. All the experiments were performed in triplicate and the concentration of antioxidants in the spiked ultrapure water samples was 500 ng mL^−1^.

### 3.1. Selection of Extraction and Dispersive Solvent

**Figure 1:**
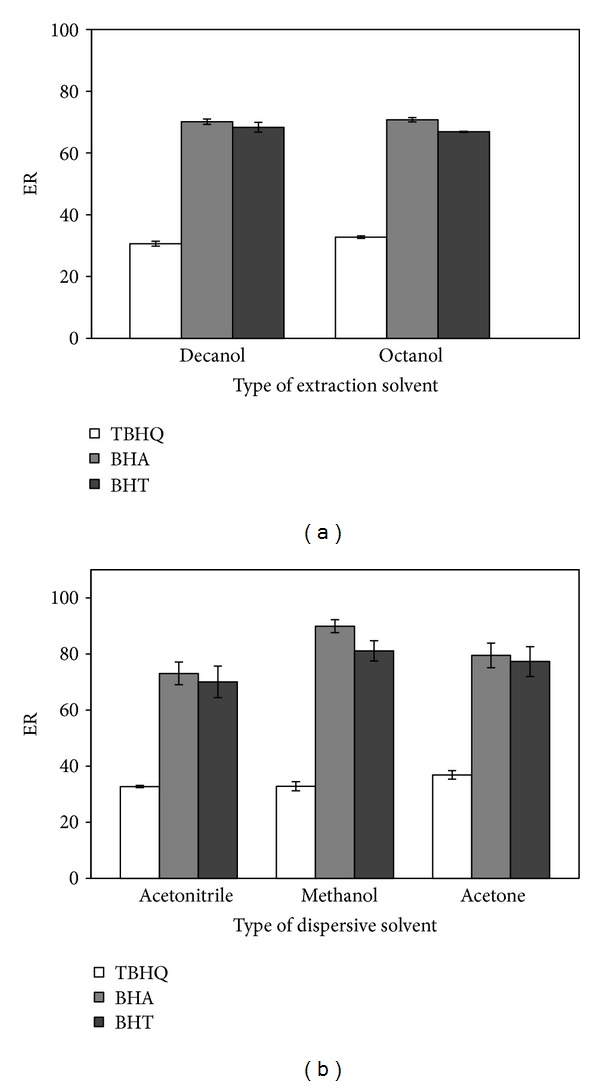
Effect of type of extraction (a) and dispersive solvent (b). Extraction solvent volume, 50 *µ*L; dispersive solvent volume, 1 mL; sample volume, 5 mL; spiked concentration, 500 ng mL^−1^.

Selection of an appropriate extraction solvent is of great importance in a DLLME process. The solvent should have good chromatographic behavior under the selected HPLC conditions, higher/lower density than water, high capability to extract the interesting analytes, and low solubility in water. 1-octanol (density, 0.82 g/mL) and 1-decanol (density, 0.83 g/mL) were tested for the extraction of selected antioxidants from aqueous sample.  Figure 1 shows the effect of extraction solvent type on the extraction efficiency. The experiments were carried out by using 1 mL of acetonitrile containing 50 *μ*L of extraction solvent. Under these experimental conditions, the volumes of the collected phase were 46 ± 1 for 1-decanol and 43 ± 2 for 1-octanol. The results indicated that the organic solvents exhibited similar extraction efficiencies for analytes, but 1-octanol has slightly higher extraction recoveries (33–71%) in comparison with 1-decanol. Therefore, 1-octanol was selected as the extraction solvent in the subsequent experiment.

In the DLLME, the miscibility of the extraction solvent with the aqueous sample is the main criterion for selecting the disperser solvent. The disperser solvent should be soluble in the extraction solvent and miscible in the water, thus, enabling the formation of fine droplets of the extraction solvent in the aqueous phase [27]. Therefore, acetonitrile, methanol, and acetone were tested as dispersive solvents and the effect of these solvents on the performance of DLLME was investigated. A series of sample solutions were examined using 1 mL of each of the disperser solvents containing 50 *μ*L of 1-octanol (Figure 1). Extraction recoveries of each antioxidant ranged between 33 and 73% for acetonitrile, 33 and 90% for methanol, and 37 and 80% for acetone. It was clear that the best extraction efficiency was obtained when methanol was used as a disperser solvent. Hence, the subsequent experiments were performed using methanol as the disperser solvent.

### 3.2. Effect of Volumes of Extraction and Dispersive Solvent

**Figure 2:**
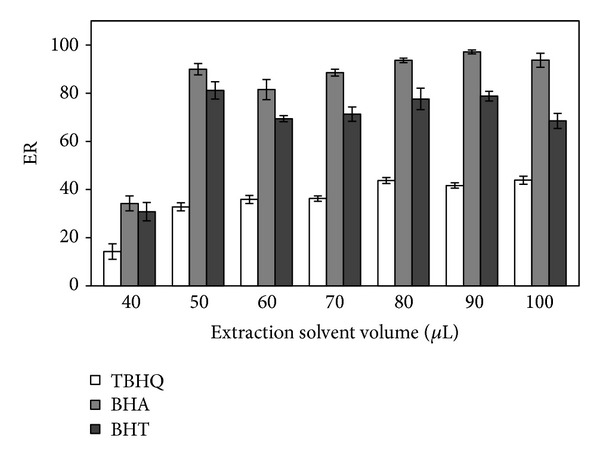
Effect of volume of extraction solvent. Dispersive solvent volume, 1 mL; sample volume, 5 mL; spiked concentration, 500 ng mL^−1^.

Optimization of volumes of the extracting solvent and the dispersing solvent is a further step in development of a DLLME procedure. Both of these volumes can influence formation of dispersion and thus have to be optimized. In order to study the effect of extraction solvent volume on the extraction efficiency, different volumes of 1-octanol (40–100 *μ*L in 10 *μ*L intervals) and a constant volume of dispersive solvent (methanol, 1 mL) were tested. By increasing the volume of the extraction solvent (1-octanol) from 40 to 100 *μ*L, the volume of the collected phase increased from 30 to 96 *μ*L. It was observed (Figure 2) that the extraction recoveries were increased by increasing the 1-octanol volume up to 90 *μ*L for TBHQ, BHA, and BHT from 14, 34, and 31% to 42, 97, and 79%, respectively. Extraction recoveries were almost constant above 90 *μ*L. The enrichment factor of the analytes decreased by increasing the volume of 1-octanol, which was an expected result due to dilution of the extracted analytes in the extraction solvent at higher volumes. Therefore, 90 *μ*L of 1-octanol was selected in order to obtain high recoveries and relatively high enrichment factors. 

**Figure 3:**
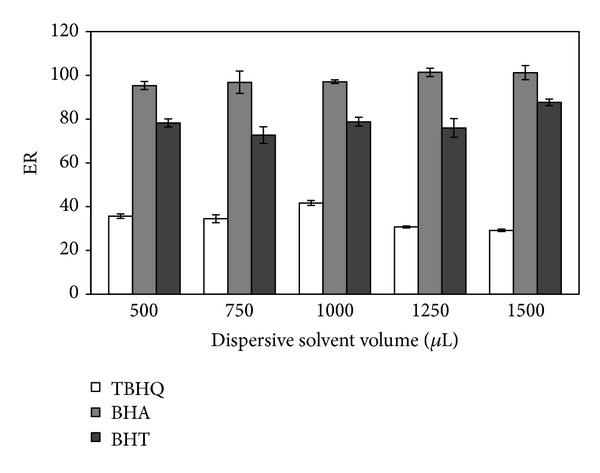
Effect of volume of dispersive solvent. Extraction solvent volume, 90 *μ*L; sample volume, 5 mL; spiked concentration, 500 ng mL^−1^.

The effect of the dispersive solvent volume was tested for five volumes of methanol (0.50, 0.75, 1.00, 1.25, and 1.50 mL). The extracting solvent volume was kept constant at 90 *μ*L. The results are presented in Figure 3. It was observed that there was no considerable change in the recovery of most antioxidants by increasing the volume of methanol from 0.50 to 1.50 mL. The prevailing view in the literature is that the cloudy state is not fully formed at low volumes of the dispersive solvent, whereas at higher volumes the solubility of the analytes in aqueous samples increases; therefore, the extraction efficiencies decrease [16, 19, 28]. Consequently, 1.00 mL was chosen as the optimum volume of the dispersive solvent.

### 3.3. Effect of pH

**Figure 4:**
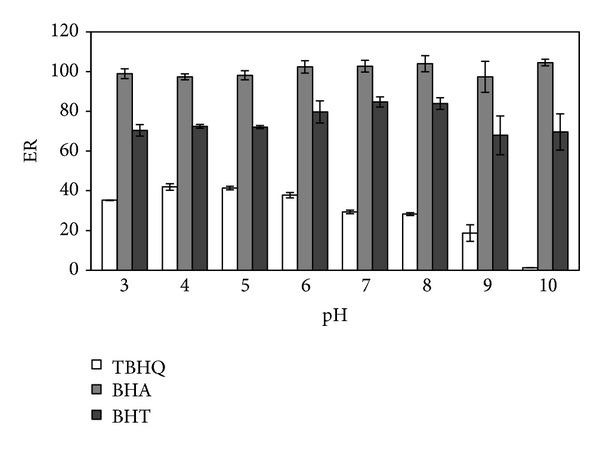
Effect of pH. Extraction solvent volume, 90 *μ*L; dispersive solvent volume, 1 mL; sample volume, 5 mL; spiked concentration, 500 ng mL^−1^.

Sample pH is another important parameter that might affect the extraction efficiency, because the analytes will be present at different forms at different pH conditions. A series of experiments were performed to investigate the effect of pH on the DLLME of antioxidants by adjusting the pH of the samples over the range of 3–10 with 0.1 mol L^−1^ NaH_2_PO_4_ and drop by drop addition of 0.1 mol L^−1^ HCl or 0.1 mol L^−1^ NaOH. It is well known that the pH of the sample solution should be lower than pK_*a*_ of the analytes for keeping analytes in their undissociated forms. TBHQ, BHA, and BHT have pK_*a*_ values higher than 10 [1]. As shown in Figure 4, the extraction recovery of TBHQ remained nearly constant over the pH range from 3.0 to 6.0, but significantly decreased as the pH increased from 6.0 to 10.0. For BHA and BHT, no obvious variation in extraction recoveries was observed until the pH was raised to 10. The maximum extraction efficiency of DLLME was achieved at pH value of 6.0, in which the analytes are completely in their molecular form. Hence, the pH of the sample solution was adjusted to 6.0 for subsequent extractions.

### 3.4. Effect of Ionic Strength

**Figure 5:**
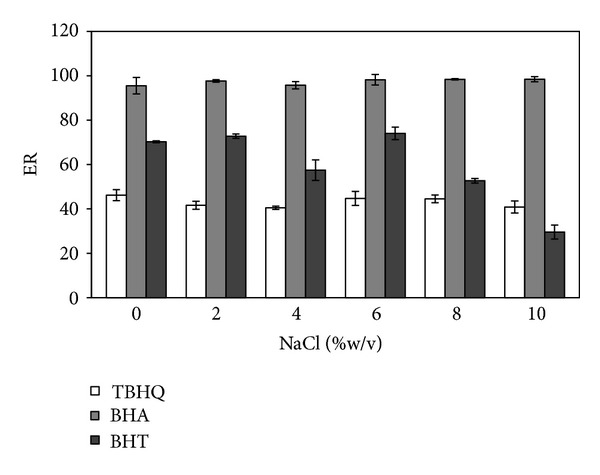
Effect of ionic strength. Extraction solvent volume, 90 *μ*L; dispersive solvent volume, 1 mL; pH, 6; sample volume, 5 mL; spiked concentration, 500 ng mL^−1^.

Generally, adding a salt decreases the solubility of analytes in the aqueous phase and enhances their extraction into the organic phase [29]. To assess the influence of ionic strength on extraction efficiency of DLLME, a series of experiments were performed by increasing NaCl concentration in the range of 0–10% (w/v) at an interval of 2% in the sample solution. With the increase of the content of NaCl from 0 to 6%, no significant effect on extraction efficiency was observed (Figure 5). Further increase of salt concentration up to 10% caused a gradual decrease in the extraction recovery of BHT. At 6% NaCl concentration, extraction efficiency of the analytes reached its maximum level; thus, subsequent experiments were carried out in the presence of 6% NaCl.

### 3.5. Effect of Extraction Time

**Figure 6:**
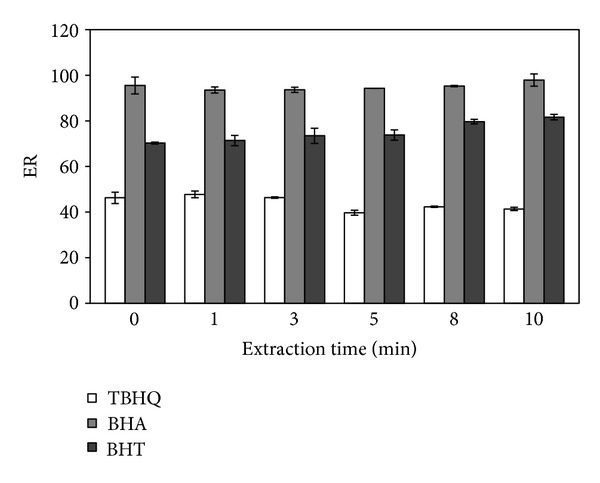
Effect of the extraction time. Extraction solvent volume, 90 *μ*L; dispersive solvent volume, 1 mL; pH, 6; ionic strength, 6% (NaCl, w/v); sample volume, 5 mL; spiked concentration, 500 ng mL^−1^.

The effects of the extraction time on DLLME of the analytes were investigated. After the addition of the mixture of 1-octanol and methanol, the sample solution was shaken by a vortex mixer for a series of extraction times in the range of 0–10 min with constant experimental conditions. The experimental results are presented in Figure 6. The results demonstrated that the extraction time had no significant effect on extraction efficiency. It was revealed that transfer of analytes from aqueous phase to extraction solvent was very fast due to the considerably large surface area between the aqueous phase and the extraction solvent. This is the most important advantage of DLLME technique [16, 17]. Thereby, additional extraction time was not required. The mixture was shaken gently for a few seconds prior to centrifugation.

### 3.6. Analytical Performance of the Proposed Method

The proposed method was evaluated under the optimized condition for the linear range, limits of detection (LOD), limits of quantification (LOQ), repeatability, enrichment factor, and extraction recovery. The results are summarized in Table 1. Linearity was observed in the ranges of 0.05–1 *μ*g mL^−1^ for BHT and 0.005–1 *μ*g mL^−1^ for TBHQ and BHA, with the square of correlation coefficients (*R* ^2^) ranging from 0.9975 to 0.9997. The limits of detection (LOD), based on a signal-to-noise ratio (S/N) of 3, ranged from 0.85 to 2.73 ng mL^−1^. The repeatability was studied for three replicate analyses of the spiked samples at a concentration level of 0.5 *μ*g mL^−1^ of each antioxidant. The relative standard deviations (RSDs) were satisfactory, ranging from 1.0% to 5.2% for all compounds, showing the good repeatability of the method. The enrichment factors and extraction recoveries were ranged between 31 and 55 and 53 and 96%, respectively.

### 3.7. Comparison of the Proposed DLLME Method with Other Methods

The efficiency of the proposed DLLME method was compared with the previously reported methods employed for the determination of antioxidants in aqueous samples. The considered parameters were sample volume, extraction time, linear range, limits of detection, and relative standard deviation. The details of the comparison are summarized in Table 2. In respect of other methods, the proposed method has a very short extraction time due to the large surface area formed between the extraction solvent and the sample solution. The proposed method has LOD better than or comparable with those of other extraction methods. It should be noted that in some of the techniques mentioned [14, 15], large volume water samples were used which inherently increased sensitivity. The proposed method also gives better or similar performance among all the other parameters compared. By considering the results, this method proved to be a rapid, sensitive, repeatable, and easy to use technique in the determination of antioxidants in aqueous samples. 

### 3.8. Sample Analysis

**Figure 7:**
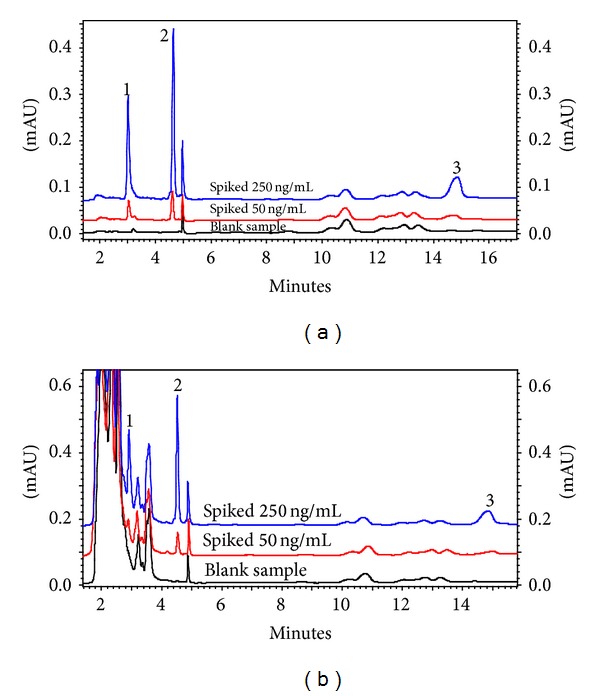
HPLC-UV chromatograms of antioxidants at the concentration levels of 50 and 250 ng mL^−1^in mineral water (a) and cherry juice sample (b) before and after spiking. Extraction solvent volume (1-octanol), 90 *μ*L; dispersive solvent volume (methanol), 1 mL; pH, 6; ionic strength, 6% (NaCl, w/v); sample volume, 5 mL. Peak assignment: (1) TBHQ, (2) BHA, (3) BHT.

The proposed analytical method was applied to determine three synthetic antioxidants in beverage samples. Different matrices of samples were studied including tap water, mineral water, cherry juice, apple juice, and mixed fruit juice. Recovery experiments were performed at spiked concentration levels of 50 and 250 ng mL^−1^ by adding the standard solution into the undiluted beverage samples. For each sample, the extraction was repeated three times. Relative recoveries and relative standard deviations were calculated and listed in Table 3. The results indicated that the samples were free of antioxidants. As can be seen, recoveries were in the range of 78–102% for all analytes in the spiked samples indicating that the real water matrices had almost little effect on the extraction efficiency, and the method could be used for the determination of synthetic antioxidants in beverages.  Figure 7 shows typical chromatograms of the mineral water and cherry juice samples spiked at the concentration levels of 50 and 250 ng mL^−1^ after DLLME.

## 4. Conclusions

In the present study, a DLLME technique combined with HPLC-UV has been proposed for the rapid and sensitive determination of three synthetic antioxidants (TBHQ, BHA, and BHT) in beverage samples. The proposed method yielded acceptable relative recoveries (78–102%) and good repeatabilities (1.0–5.2%). Moreover, high sensitivity with LODs between 0.85 and 2.73 ng mL^−1^ was achieved by using a sample volume of only 5.0 mL. Based on these advances, the proposed method could be used for monitoring as well as controlling the synthetic antioxidants in aqueous food samples.

## Figures and Tables

**Table 1:** Analytical performance of the proposed method for the determination of antioxidants.

Analyte	LR^a^ (*µ*g mL^−1^)	R^2^ ^b^	LOD^c^ (ng mL^−1^)	LOQ^d^ (ng mL^−1^)	RSD^e^ (%)	EF ± SD^f^	ER% ± SD^g^
TBHQ	0.005–1	0.9980	0.85	2.82	1.0	31 ± 1	53 ± 1
BHA	0.005–1	0.9997	1.67	5.56	2.2	55 ± 2	96 ± 3
BHT	0.05–1	0.9975	2.73	9.09	5.2	40 ± 3	70 ± 5

^a^Linear range.

^b^Square of correlation coefficient.

^c^Limits of detection (*S*/*N* = 3).

^d^Limits of quantification (*S*/*N* = 10).

^e^Relative standard deviation (*C* = 0.5 *µ*g mL^−1^, *n* = 3).

^f^Mean enrichment factor ± standard deviation (*n* = 3).

^g^Mean extraction recovery ± standard deviation (*n* = 3).

**Table 2:** Comparison of the proposed DLLME method with other methods used in determination of antioxidants.

Method	Sample	Volume	Analytes	Extraction time (min)	LR (*µ*g L^−1^)	*R* ^2^	LOD (ng mL^−1^)	RSD (%)	Ref.
SPME-GC-MS^a^	Drinking water	15 mL	BHT	30 min	12.8–64.0	0.998	4.2	7–14	[30]

SPE-GC-MS^b^	River water	5 L	BHT	—	—	—	0.001	6	[15]

O-CLLE-GC-MS^c^	Effluent samples	40 L	BHA BHT	<23 min	0.025–1 0.0125–0.5	0.994 0.993	0.01 0.008	19.7 34.3	[14]

SPE-GC-MS	Aqueous samples	200 mL	TBHQ BHA BHT	—	2–2000 2–2000 2–2000	0.996 0.994 0.997	0.03 0.8 0.2	2 3 4	[1]

DLLME-HPLC-UV^d^	Fruit juice	40 mL	BHA BHT	10 min	10–2500 2–2500	0.9993 0.9989	2.5 0.9	2.7 4.2	[9]

DLLME-HPLC-UV	Beverages	5 mL	TBHQ BHA BHT	<1 min	5–1000 5–1000 50–1000	0.9980 0.9997 0.9975	0.85 1.67 2.73	1.0 2.2 5.2	This study

^a^Solid-phase microextraction-gas chromatography-mass spectrometry.

^b^Solid-phase extraction-gas chromatography-mass spectrometry.

^c^Online continuous liquid-liquid extraction-gas chromatography-mass spectrometry.

^d^Dispersive liquid-liquid microextraction high-performance liquid chromatography-ultraviolet detection.

**Table 3:** Spiked recoveries of antioxidants in beverage samples by the proposed method.

Sample	Added (ng/mL)	TBHQ		BHA		BHT	
		Found ± SD (ng/mL)	RR (%)	Found ± SD (ng/mL)	RR (%)	Found ± SD (ng/mL)	RR (%)
Tap water	50	47.3 ± 2.2	95	47.8 ± 1.3	96	44.3 ± 4.6	89
	250	237.7 ± 9.8	95	244.1 ± 8.3	98	245.8 ± 4.8	99
Mineral water	50	50.9 ± 1.2	102	50.1 ± 2.5	100	49.2 ± 1.3	98
	250	240.8 ± 2.7	96	241.4 ± 6.0	97	247.1 ± 12.3	99
Cherry juice	50	47.7 ± 3.0	95	39.9 ± 2.9	80	43.4 ± 2.4	87
	250	254.5 ± 8.9	102	241.6 ± 6.8	97	240.9 ± 6.7	96
Apple juice	50	44.2 ± 2.4	88	46.2 ± 1.5	92	46.8 ± 2.9	94
	250	213.4 ± 10.2	85	233.8 ± 11.6	94	241.7 ± 8.1	97
Mixed fruit juice	50	39.1 ± 2.5	78	46.1 ± 1.1	92	43.8 ± 2.6	88
	250	205.6 ± 8.8	82	240.3 ± 6.9	96	244.4 ± 7.7	98

RR: relative recovery.

SD: standard deviation (*n* = 3).
